# An algorithm for differentiating food antigen-related gastrointestinal symptoms 

**Published:** 2021

**Authors:** Kamran Rostami, Justine Bold, Jafer Ismail Ali, Alison Parr, Walburga Dieterich, Yurdagül Zopf, Aung Htoo, Mohammad Rostami-Nejad, Mihai Danciu

**Affiliations:** 1 *Department of Gastroenterology, MidCentral DHB, Palmerston North, New Zealand *; 2 *Department of Gastroenterology, Milton Keynes University Hospital NHS Trust, UK*; 3 *The School of Allied Health and Community, University of Worcester, Worcester, UK*; 4 *Centre for Medical Education, School of Medicine, Cardiff University, Cardiff UK*; 5 *Salford Community Leisure, Manchester, UK*; 6 *Department of Medicine 1, Friedrich-Alexander-Universität Erlangen-Nürnberg, Germany; Hector Centre of Excellence for Nutrition, Exercise and Sports, Friedrich-Alexander-Universität Erlangen-Nürnberg, Germany*; 7 *Gastroenterology and Liver Diseases Research Center,* *Research Institute for Gastroenterology and Liver Diseases, Shahid Beheshti University of Medical Sciences, Tehran, Iran*; 8 *Grigore T Popa” University of Medicine and Pharmacy, Iasi, Romania*

**Keywords:** NCGS, Gluten, Fructans, Amylase trypsin inhibitors, FODMAP, Lactose intolerance, Irritable bowel syndrome

## Abstract

**Aim:**

The aim of this clinical audit was to assess patient-reported outcomes on the effect of dietary intervention, to enhance our understanding of possible treatment options in irritable bowel syndrome (IBS).

**Background:**

A large number of food-related gastro-intestinal disorders have been attributed to IBS for decades.

**Methods:**

Patient-reported outcomes from the records of 149 IBS patients treated at secondary and tertiary Gastroenterology outpatients in two UK hospitals between January 2014 and July 2016 were audited. Patients all presented with symptoms fulfilling Rome III-IV criteria for IBS had negative coeliac serology and did not have other gastrointestinal (GI) conditions. A modified version of a low FODMAP diet had been recommended (gluten and lactose free diet (G/LFD)) and was implemented for 6 weeks. Outcomes and dietary adherence were recorded during outpatient’s consultations.

**Results:**

A total of 134 patients complied with the diet optimally. The majority had an improvement rate >70% and continued with the diet. Fifty-three percent became completely or almost asymptomatic, while 27.6% had a poor response to the diet (scoring < 30%) to G/LFD. The improvement was excellent in patients with normal BMI and good in overweight and obese and where BMI <18. Over 50% did not require any follow-up within 12 months.

**Conclusion:**

Although it is unclear whether symptoms are triggered by gluten, fructans or lactose, elimination of gluten and lactose proved to be an effective treatment in patients with IBS. Multidisciplinary team management and implementation of detailed nutrition therapy using the audit algorithm might prove to be both cost effective and efficacious a treatment option in IBS.

## Introduction

 Irritable bowel syndrome (IBS) is a collection of gastrointestinal symptoms that was defined 4 decades ago when no organic causes were identified for the symptoms. Due to multiple etiologies, the pathogenesis of IBS is poorly understood. 

The Manning criteria were originally developed in 1978 (1), followed by the Rome criteria in 1992. The Rome Criteria have been periodically revised (Rome IV criteria) to enable the health professional to filter the functional from organic disorders (2). Both Manning and Rome criteria have been criticized for their low specificity. In recently published Rome IV criteria, some of previously considered functional conditions have been removed from the IBS box (3) as a clear pathophysiology, and a distinct immunopathology were identified for these conditions. This has most likely contributed to a recently reported reduced incidence of IBS (4).

Discovery of foods high in FODMAP (including wheat and milk products) as a triggering factor for some IBS patients has revolutionized our understanding on etiopathogenesis of IBS. It has explained that food sensitivity triggers symptoms in a substantial number of patients under IBS umbrella (5). Studies demonstrate that many patients with IBS benefit from a low FODMAP diet (6). However, the long-term implications of following a low-FODMAP diet are poorly understood and there is a risk of both poor diet quality (7) and microbiome harm (8). Wheat is high in fructans and is a staple food in many cultures; thus, exclusion of wheat and gluten-containing foods can be a simpler way of reducing FODMAPs without wider dietary restriction of all FODMAP-rich foods, many of which are fruits and vegetables. Prior to the discovery of food implications in IBS, patients presenting with gastrointestinal symptoms who were compatible with Rome I-IV criteria were treated only symptomatically with medications without exploration of the underlying cause of symptoms. The symptom-control approach has been associated with patients’ dissatisfaction, additional anxiety and psychological consequences resulting from experiencing unresolved and persistent symptoms without a clear medical explanation. The downside of symptomatic treatment is not limited to patients’ dissatisfaction, rather it lacks long-term efficacy (9). Moreover, the side effects of medications, the ongoing investigations and outpatient visits exhaust health-organization resources (10) and impair the quality of life of patients. 

There are several randomized controlled trials (11-14) suggesting that a large proportion of patients presenting with IBS symptoms would respond to dietary intervention, gluten-free diet (GFD) in particular. In these studies, quantities of gluten were used for challenge purposes (between 3-52g/day). 

Therefore, current evidence demonstrates that a large proportion of these patients can be treated effectively with a simpler version of low FODMAP comprising principally of a GFD. In addition, lactose intolerance is often a missed diagnosis (especially prevalent in multi-cultural communities) and some patients with this condition eventually receive medication instead of having lactose eliminated (15). 

The aim of this audit was to assess the outcomes achieved using a lactose and GFD clinical intervention in patients traditionally diagnosed with IBS. 

## Methods

This project was registered and approved by research development & audit department of both Worcester Acute and Milton Keynes University Hospitals (with registration number 993). This was an audit of outcomes from the records of 149 patients presenting with IBS symptoms at secondary and tertiary Gastroenterology outpatients in two UK hospitals between January 2014 and July 2016. All patients were presenting with symptoms consistent with Rome III-IV criteria. Organic disorders were excluded in patients with red flag signs like anaemia and weight loss via screening for coeliac disease or other gastrointestinal conditions. Lifestyle advice was given to overweight and obese patients to avoid hyperphagia by eating moderate portion sizes and ensuring adequate mastication. Patients were then offered a dietary intervention consisting of a gluten and lactose free diet (GF/LFD) for six weeks. Demographics, presenting symptoms, and serologic and histologic data were recorded. Clinical evaluation was performed using a self-administered instrument based on patient declaration during their outpatient’s consultations. Extra-intestinal Non Coeliac Gluten Sensitivity (NCGS) manifestations were recorded. The patients identified one to three main symptoms that were quantitatively assessed using a Numerical Rating Scale (NRS) with a score ranging from 1 (mild) to 10 (severe) (16). The response was assessed for each parameter separately. A symptomatic response was a decrease of at least 30% of the baseline score. Responders were defined as patients who fulfilled the response criteria (> 30% reduction of one to three main symptoms or at least 1 symptom with no worsening of others).

Dietary adherence was evaluated during their follow-ups. Patients were instructed and monitored by dietitians and gastroenterologists. Following dietary exclusion, a diagnosis of NCGS was made in some cases based on Salerno expert criteria (16) (however, as this was in a hospital outpatient clinical environment, an open gluten challenge was used instead of randomised double-blind placebo-controlled gluten challenge). 

**Statistical analysis**

Data were expressed as mean ± standard deviation for numeric data and frequency (percent) for categorical data. Data were compared regarding different body mass indexes (BMI). Chi- square test, or alternatively Fisher test, was used for categorical data. P-value of less than 0.05 was considered as significant.

## Results

A total of 134 out of 149 patients followed a Gluten- and lactose-free diet (G/LFD) and complied with the diet optimally. Fifty-six patients (41.8%) were from Milton Keynes University Hospital and 78 (58.2%) from Worcester Acute Hospitals. A number of patients were tertiary referrals included in both centres. The ages of patients ranged from 8 to 85 years, with a mean age of 46.41 + 17.388 years. The majority (109) were females (81.3%), while the number of male patients was 25 (18.7%).

As much as 72.4% (97/134 cases) showed significant improvement with a score in the range of 40-100% (P=0.001), while 27.6% had a poor response with a score < 30%. From the group of responders, 30/97 (32%) became completely asymptomatic. The improvement reported in the rest of responders (67/97) scored between 40-95%. Over 50% of the patients did not require a further follow-up within next 12 months owing to improvements in symptoms. 

**Figure 1:**
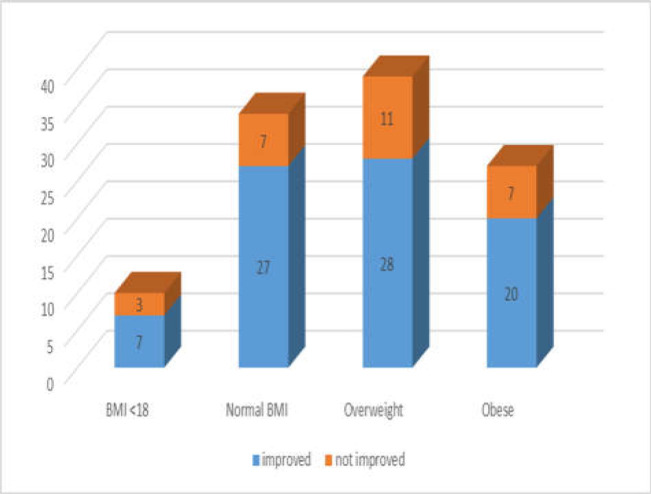
Body mass index and response to elimination diet (GF/LFD)

**Figure 2:**
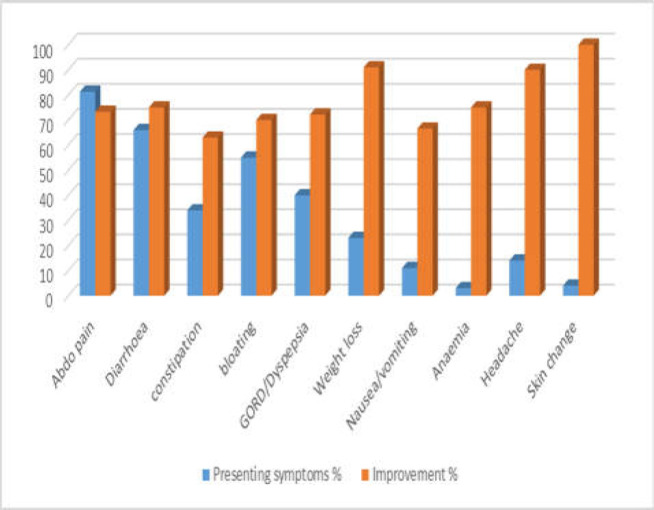
Symptoms and response to GF/LFD

In 110 patients, body mass index (BMI) was measured and from this group 10 (9 %) patients had low BMI, 34 (31%) normal BMI, 39 (35.5%) were overweight and 27 (24.5%) were obese. The best response to elimination diet was achieved in 27 cases with normal BMI followed by 28/39 in overweight range. There were no significant differences between response to elimination diet in patients with lower BMI <18 or obese (figure 1). The frequency of symptoms and response to GFD is summarized in Figure 2.

**Figure 3:**
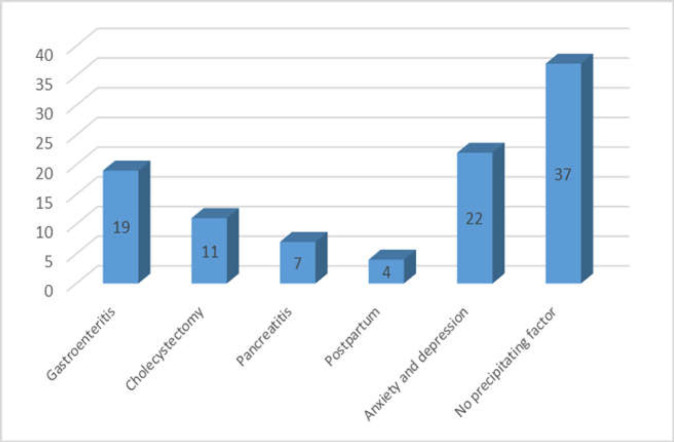
Reported precipitating factors in the study population (%)

**Table 1:** The frequency of symptoms and response to the GF/LFD

Symptoms	Frequency (%)	Response to GFD (%)
Abdominal pain	109 (81.3)	80/109 (73.3)
Diarrhoea	88(66)	66/88 (75)
Constipation	46 (34)	29/46 (63)
Bloating	73 (55)	51/73 (70)
Reflux/Dyspepsia	54 (40)	39/54 (72.2)
Weight loss	31 (23)	21/31 (91.3)
Nausea/vomiting	15 (11)	10/15 (66.6)
Anaemia	4 (3)	3/4 (75)
Headache	19 (14)	17/19 (90)
Skin change	6 (4)	6/6 (100)

The most prevalent GI symptoms were abdominal pain at 109/134 (81.3%) followed by diarrhoea 88/134 (66%), bloating 73/134 (55%), and heartburn 54/134 (40%). The rate of improvement for abdominal pain scored as high as in 80/109 similar to diarrhoea in 66/88 and bloating in 51/73. (Table 1) Surprisingly dyspeptic symptoms also improved in 39/54 (72.2%) in a similar range like diarrhoea and abdominal pain. (Table 1) A number of patients were able to stop or reduce taking their proton pump inhibitor (PPI) medication. Despite the restrictive nature of diet, 21/31 with weight loss gained or maintained their weight. It should therefore be acknowledged that nutritional deficiency is common in patients with non-coeliac gluten-related disorders (17). Significant improvement was also reported in 10/15 patients with nausea and vomiting, 17/19 with headache, and 6/6 with skin changes (See Figure 2). The triggering factors were assessed in 110/134 patients. In 62.3% of this group, we found a range of precipitating factors that included post gastroenteritis in 21/110 (19%), post cholecystectomy in 12/110 (11%), post pancreatitis in 8/110 (7%), postpartum in 4/110 (3.6%), and anxiety and/or depression 24/110 (22%). Nevertheless, for 41/110 (37%) there were no identifiable triggering factors identified (See Figure 3).

## Discussion

IBS-like symptoms account for 40–60% of referrals to gastroenterology outpatient clinics (18). Prescribing analysis and cost tabulation (PACT) in the UK indicated that more than £70,000,000 has been spent on selected new laxatives and antispasmodics commonly used to treat IBS in primary care during 2012-2013 (10). When patients are diagnosed and treated in secondary care, the total healthcare costs per patient substantially increases from 486 Euro (±3192) to 2328 Euro (±5888) according to a Dutch study (19). Similarly, the average total direct medical cost/patient/year is estimated at USD 1.35 billion in the USA and 756.14±1592 euros per patient in France (20). The results of this clinical audit suggest that many patients of this group could potentially be managed more cost-effectively with dietary therapy.

**Figure 4:**
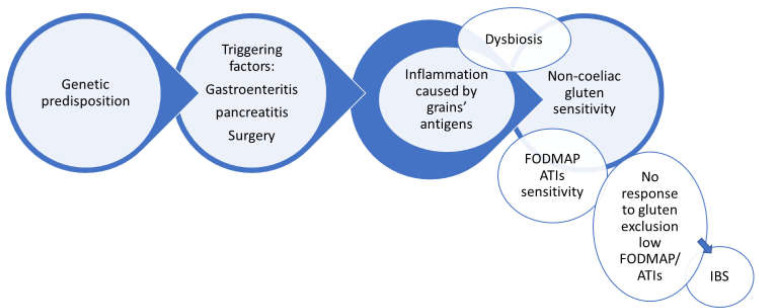
Triggering factors and pathomechanism

**Figure 5:**
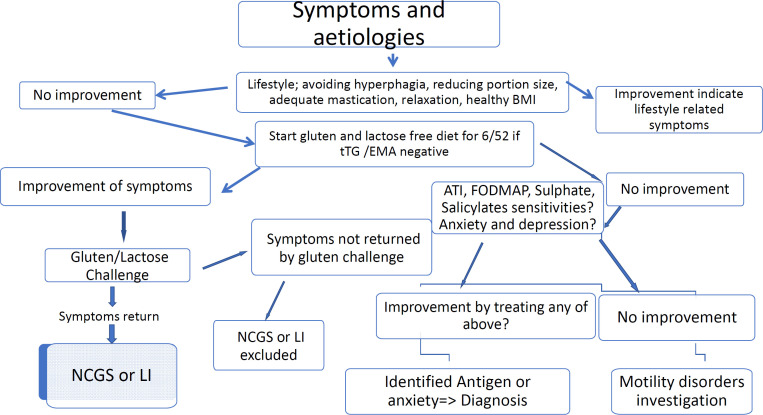
Algorithm to differentiate Gluten/Lactose//Fructans/ATIs sensitivity from IBS. LI=lactose intolerance. Six-week gluten- and lactose-free diet followed by one-week gluten challenge. When NCGS has been diagnosed or excluded, a 7-day Lactose challenge will follow to exclude or ascertain lactose intolerance

This audit has demonstrated that more than 70% of patients presenting with IBS symptoms improved by following a diet eliminating lactose and gluten containing grains (improvement for >30% in their symptoms). The variable response to dietary intervention suggests a multifactorial etiology to food sensitivity. The spectrum of variable responses to the gluten containing grain exclusion would suggest the possible overlapping (21) of other food antigens as outlined in figures 4 and 5. There was 40-95 % improvement in symptoms following elimination diet in 53% of our patients, which suggests sensitivity to gluten or other component confined to gluten containing grains or lactose. The lesser improvement rate might be associated with other factors like inadequate compliance with exclusion diet, possible implication of fructans (22) or anti-trypsin inhibitor (ATI) sensitivity. 

A diagnosis of IBS was applicable to 18% of patients included in this audit who had 0% response to elimination of gluten and lactose. Nevertheless, a comprehensive additional full FODMAP and ATI exclusion would be practically needed to be undertaken before a definite diagnosis of IBS is made in non-responsive patients to gluten and lactose exclusion. 

The success of the elimination diet did not seem to be correlated with the body mass index (BMI). The best outcome was recorded in patients with normal BMI and also in the overweight group. Patients with higher BMI >30 or low below 18 also responded well to nutrition therapy.

Based on this finding and spectrum of improvement, we proposed an algorithm in which food sensitivity could be differentiated from IBS. In this algorithm, gluten- and lactose-free diet stand as the first line of elimination intervention for patients presenting with IBS symptoms. This strategy is much less restricted compared to low FODMAP pathway with a success rate in this audit of 72%. For those with lower improvement score, a full low FODMAP / ATI should be considered as the second line. Symptomatic treatment with medicines might be best considered in those who do not wish to undergo or respond to the elimination diet (See Figure 5 Algorithm).

**Figure 6:**
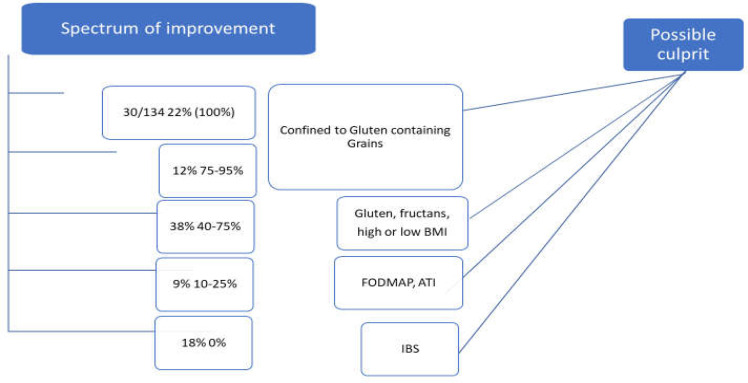
Spectrum of improvement and correlation with possible culprit

From a patient’s perspective, lack of a good explanation for symptoms may cause additional anxiety and depression (23, 24) so having an understanding of the cause of symptoms can also be beneficial. 

The symptoms related to non-coeliac gluten or fructans sensitivity are nearly identical to those of lactose intolerance or ATI related. Lactose intolerance (LI) in particular and secondary LI are common and underestimated. Despite the strong evidence suggesting high prevalence of this condition , a large proportion of both primary and secondary lactose intolerance are underestimated, labelled and treated like IBS with medications. Dietary advice is rarely provided by Gastroenterologists even though the impact of food-related disorders compromise such high proportion of Gastroenterology outpatients. Astonishing 79% of Native Americans, 75% of African Americans, 51% of Hispanics, and 21% of Caucasians are reported to suffer from lactose maldigestion (25). In Africa, Asia, and Latin America, prevalence rates vary in the range 15-100% depending on the population studied (25). Lactose, fructans and galacto‐oligosaccharides have strong biologic plausibility for symptom generation due to lack of hydrolases resulting in distention from osmosis and rapid fermentation (26). Lactose, gluten and other components of grains like ATIs are another major culprit for symptoms in a large proportion of patients fulfilling diagnostic criteria of IBS. Wheat proteins have been reported to dysregulate the gut function (27) as antigenic wheat proteins activate innate lymphoid cell population (28) resulting in epithelial cells damage (29). They also lead to state of sensitivity with coeliac-like intestinal and extra-intestinal symptoms (30) that may present with mild or often invisible enteropathy in susceptible individuals (31).

It is, however, unclear which component of grains are principal causes for these symptoms. Gluten (32), fructans (33) and ATIs (34) have been reported as major antigens in this equation. As far as we know, there is no published study that reliably demonstrates an exclusive antigenic property of any of these 3 grains’ components (35). They may not be mutually exclusive either, as some individuals could potentially have sensitivity to both gluten and FODMAP. 

In accordance to some studies, excess fructose and polyols may only cause symptoms in specific individuals when consumed in high doses (26). 

The results of this audit are similar to a number of previous RCTs; most of the patients’ symptoms improved by avoiding gluten containing grains and lactose. The identification of the underlying cause for IBS symptoms was out of the scope of this audit, however. 

The clinical team reported that gluten-containing grains proved to be the main factor behind the symptoms of the majority of this group of patients. Nevertheless, evidence to prove this is not available from the audit as the clinical intervention included the exclusion of both lactose and gluten. In addition, it is impossible to identify from the records if it was the exclusion of lactose, gluten or indeed the other components of grains or perhaps a combination of these factors that provided the main benefit. 

**Triggering factors**

The reason why the food sensitivity occurs at different stages of people’s lives has been a matter of debate. 

Environmental factors including an alteration of the gut microbiota (35) may be associated with NCGS and secondary lactose intolerance, but it is unclear whether dysbiosis is a primary or secondary event in the genesis of NCGS. The gut microbiota may change in patients following the events such as birth, infections, pancreatitis and surgeries (36, 37). These were found to be potential precipitating factors for developing NCGS in these individuals. The origin of antigens cannot be inferred from this audit, but one can speculate antigens to have most likely originated from grain peptides. The FODMAP elements could possibly enhance the irritability component especially in patients with lower rate of improvement to gluten exclusion. The extraintestinal presentation can only be explained or induced by systemic inflammation (27, 31), which would support the potential grain antigenicity. The above complex pathophysiology would translate the environmental factors like gluten/ATI and FODMAP into an illustration (Figure 4, 5 and 6).

Why should medication and symptomatic treatment be prescribed when elimination of triggers may prove beneficial? Current guidelines such as the NICE guidelines in the UK for IBS recommend the use of medications (39), and less emphasis is currently put on the identification and elimination of triggers that may be present in the diet. In this audit, findings were similar to several RCT in that the majority of this group had an improvement rate over 70% following implementing a gluten and lactose elimination diet. 

This number and proportion of improvement is incomparable with any medications listed in NICE guideline for IBS (which provide symptomatic relief to around 50% of patients). Around 53% of the patients in this audit became completely or significantly asymptomatic. This indicates that the elimination of grains-containing gluten is an effective therapeutic intervention (9) in improving the symptoms. In this audit, improvement was not only recorded in patients with abdominal pain, diarrhoea and reflux disease, but also documented across a range of additional extraintestinal symptoms including joint pain, skin abnormalities, milder neuropathy, headache, fatigue and general well-being.

This project was an audit and was not a randomised controlled trial, hence findings need to be considered in context of this limitation. However, findings are similar to a previous randomised double-blind placebo-controlled study (13). Another limitation is that there was no differentiation between the lactose and gluten exclusion, which was not randomised, hence it is recommended that future research focus on investigating these areas. 

For all the outlined rationalization above, we would encourage healthcare and medical practice to consider differentiating food sensitivity from IBS as the treatment of these conditions are different (39). Identifying the group of patients with food sensitivity would open a prospect toward more targeted treatment that is more cost effective, with fewer side effects that could also potentially improve quality of life and patient satisfaction. New guidelines could support multidisciplinary team working, with joint dietitian and gastroenterologist clinics, for example.

Findings from this clinical audit suggest that food sensitivity particularly in gluten-containing grain and lactose play a major role in generating IBS symptoms. Food sensitivity is a treatable condition with clear pathogenesis, and we recommend that it be differentiated from IBS using the algorithm developed as a result of this audit project. Selection of the candidates for nutrition therapy based on the algorithm may help identifying individuals with a potential of optimal response to an elimination diet. 

It may be cheaper for health systems and deliver better outcomes for patients if elimination diets are used within current clinical guidelines. The algorithm (Figure 5) would prioritise nutrition therapy above using medications with significant side effect profile (9), which are costly and may not be clinically effective in all cases. In addition, it would help to prevent further expensive investigations by providing an explanation for patients’ symptoms in a large proportion of patients (findings from this audit suggest it could be as high as 72% of IBS patients).

Dietary therapies are gaining popularity, as evidence of efficacy for specific diets has emerged. By undertaking dietary interventions, patients might not be affected by the side effects of medications currently used for their symptomatic relief. Dietary therapy also has the potential to confer financial benefits to health care providers who are already overstretched in caring for these patients.
